# JAK/STAT signaling pathway inhibitors in neurodegenerative diseases: current status and future perspectives

**DOI:** 10.3389/fnagi.2026.1880374

**Published:** 2026-07-10

**Authors:** Hai-Xia Yang, Bo-Wei Su, Ya-Nan Bao, Wen-Hao Wang, Jia-Yi Lao, Hong-Yu Ji, Shuang Han, Xiao-Hui Cui, Hui-Wen Ren, Zhi-Lin Luan

**Affiliations:** 1 Department of Clinical Laboratory, The Second Affiliated Hospital of Dalian Medical University, Dalian, China; 2 Advanced Institute for Medical Sciences, Dalian Medical University, Dalian, China; 3 Dalian Key Laboratory for Nuclear Receptors in Major Metabolic Diseases, Dalian, China

**Keywords:** blood–brain barrier, JAK inhibitors, JAK/STAT signaling pathway, neurodegenerative diseases, neuroinflammation

## Abstract

Neurodegenerative diseases (NDDs) are a major public health concern characterized by the progressive loss of neurons, ultimately leading to neuronal death and causing a sustained decline in brain function or physical motor abilities. Major examples include Alzheimer’s disease (AD) and Parkinson’s disease (PD). Currently, NDDs lack effective curative methods, and their pathological process primarily involves misfolded protein aggregation, oxidative stress, and neuroinflammation. The Janus kinase/signal transducer and activator of transcription (JAK/STAT) signaling pathway, as a central hub for cytokine signaling, has recently been found to play a key role in neuroinflammation and immune regulation in NDDs. This review systematically elucidates the core mechanisms of the JAK/STAT pathway in NDDs, including the regulation of microglial and astrocytic reactivity, the impact on blood–brain barrier integrity, and involvement in energy metabolism abnormalities. On this basis, we have reviewed and evaluated various therapeutic strategies targeting this pathway, focusing on small-molecule JAK inhibitors such as baricitinib and tofacitinib, and have analyzed their mechanisms of action, preclinical efficacy, and potential side effects. In addition, this article provides a forward-looking perspective on the future research directions of the JAK/STAT pathway from the perspective of anti-neuroinflammation to promote neuroregeneration therapy, aiming to offer theoretical references and new ideas for the clinical translational research of this pathway.

## Graphical abstract

**Figure:**
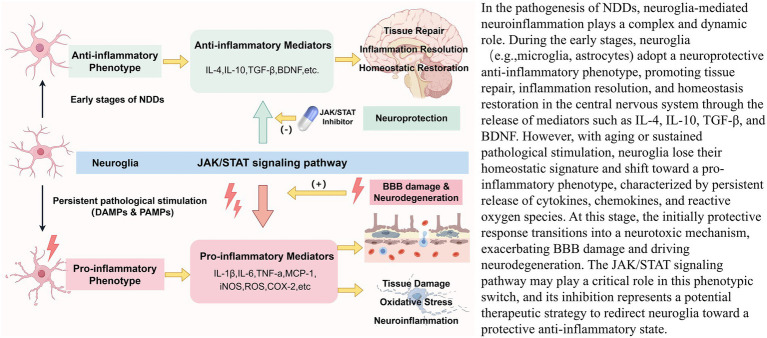

## 1 Introduction

Alzheimer’s disease (AD), Parkinson’s disease (PD), Amyotrophic lateral sclerosis (ALS), Huntington’s disease (HD), and Multiple sclerosis (MS) are currently incurable neurodegenerative diseases (NDDs), characterized by the progressive and irreversible loss of specific neuronal subpopulations within the central nervous system (CNS) (Heemels, 2016). As the global population ages, NDDs have become an increasingly severe public health issue worldwide (Chimagomedova et al., 2017). These diseases, in addition to the abnormal aggregation and accumulation of misfolded proteins (Vaquer-Alicea and Diamond, 2019), also involve many complex pathophysiological mechanisms, primarily including oxidative stress, apoptosis, abnormal autophagy, and neuroinflammation (Glass et al., 2010; Antico et al., 2025; Wang et al., 2023; Moujalled et al., 2021). However, the precise molecular pathway network driving the occurrence and development of these diseases has not yet been fully elucidated.

**Figure 1:**
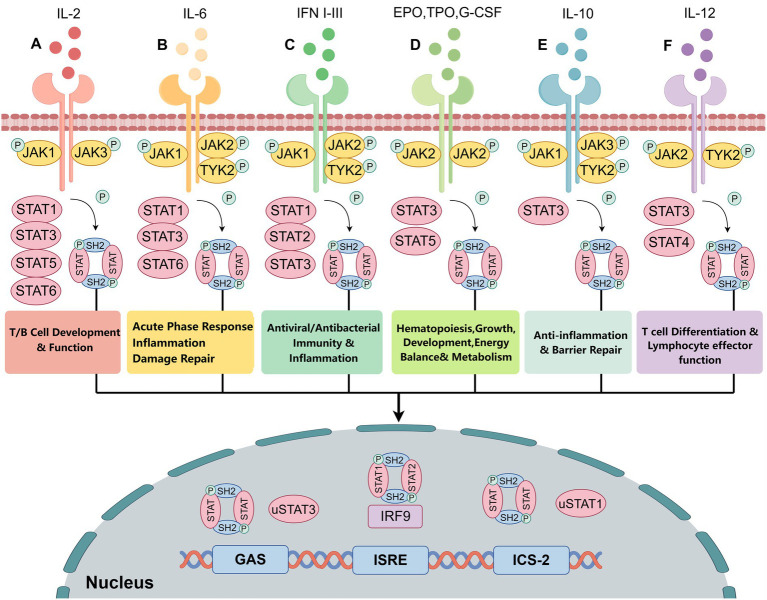
JAK/STAT signaling pathway. **(A)** Receptors for *γ*c-chain cytokines (e.g., IL-2, IL-4, IL-7, IL-9, IL-15, IL-21); **(B)** Receptors for IL-6 family cytokines (e.g., IL-6, IL-11, LIF, OSM); **(C)** Receptors for interferon family cytokines (Types I–III); **(D)** Receptors for hematopoietic growth factors (e.g., EPO, TPO, G-CSF), growth hormone/prolactin, and leptin; **(E)** Receptors for IL-10 family cytokines (e.g., IL-10, IL-22, IL-24); **(F)** Receptors for the IL-12 family cytokines (e.g., IL-12, IL-23). Canonical ligand-receptor pairs and their associated JAK and STAT isoforms. Upon receptor activation, JAKs (JAK1, JAK2, JAK3, or TYK2) undergo autophosphorylation and subsequently phosphorylate STAT proteins (STAT1–STAT6). The phosphorylated STATs then regulate gene transcription through multiple mechanisms: STAT dimers bind to Gamma-Activated Sequences (GAS); the ISGF3 complex (composed of STAT1, STAT2, and IRF9) binds to interferon-stimulated response elements (ISREs); and unphosphorylated STAT1 (uSTAT1) interacts with Interferon Consensus Sequence 2 (ICS-2) to sustain the expression of interferon-stimulated genes. Under both physiological and pathological conditions, the JAK/STAT pathway fulfills four essential roles: (1) Immune regulation: Governing T/B cell development, differentiation, and effector functions, while modulating anti-inflammatory responses and barrier repair; (2) Host defense: Mediating antiviral/antibacterial immunity, inflammatory responses, and acute phase reactions to combat infections and tumors; (3) Hematopoiesis: Controlling blood cell production and homeostasis; (4) Metabolic homeostasis: Regulating energy balance, metabolism, and growth (by Figdraw).

Against this backdrop, numerous neuroprotective signaling pathways have been proposed as potential means of slowing the progression of NDDs, with the JAK/STAT signaling pathway being one of the most closely studied. The discovery of the JAK/STAT signaling pathway originated from research on interferon (IFN)-induced transcription factor activation (Fu et al., 1990); it primarily consists of three components: the ligand-receptor complex, the signal-transducing tyrosine kinase JAK, and the effector transcription factor STAT, and is involved in the signal transduction of various cytokines and hormones, including IFN and interleukins (Thomas et al., 2015). When a ligand binds to a membrane receptor, it activates the JAK kinase associated with the receptor in the cytoplasm, which in turn phosphorylates tyrosine residues on the intracellular domain of the receptor, providing an anchor site for STAT. STAT is recruited to the receptor complex via its SH_2_ domain and phosphorylated by JAK; after forming homodimers or heterodimers, it is transported into the nucleus to regulate the expression of target genes, thereby mediating downstream events such as hematopoiesis, adaptive immunity, tissue repair, pro-inflammatory cytokine production, cell division and proliferation, cell death, and tumor formation (Owen et al., 2019; Garrido-Trigo and Salas, 2020; Villarino et al., 2020; Katz et al., 2007; Lee et al., 2011; Gadina et al., 2017; Mertens and Darnell, 2007; O'Shea and Plenge, 2012; Xin et al., 2020; Xue et al., 2023) (Figure 1).

Although the development and mechanisms of the JAK/STAT signaling pathway in NDDs remain unclear, its pivotal role in neuroinflammation and neurodegeneration is undisputed. This article aims to systematically summarize and discuss the pathogenesis of the JAK/STAT signaling pathway in NDDs and to evaluate the therapeutic potential and safety of Western medicines that target it. Furthermore, this article will explore the bridging role of the JAK/STAT pathway in linking metabolic diseases with NDDs, as well as future precision-targeting strategies, with the aim of providing a solid theoretical foundation and research perspective for the development of novel intervention strategies for NDDs.

## 2 Mechanisms of action of the JAK/STAT signaling pathway in NDDs

### 2.1 The JAK/STAT pathway mediates blood–brain barrier (BBB) dysfunction

Traditionally, it has been believed that the BBB prevents peripheral immune cells and molecules from infiltrating the CNS, thereby maintaining the CNS’s immune privilege (Muldoon et al., 2013). However, recent studies have shown that in NDDs such as AD and PD, the integrity of the BBB is often compromised (Chen T. et al., 2024).

**Figure 2:**
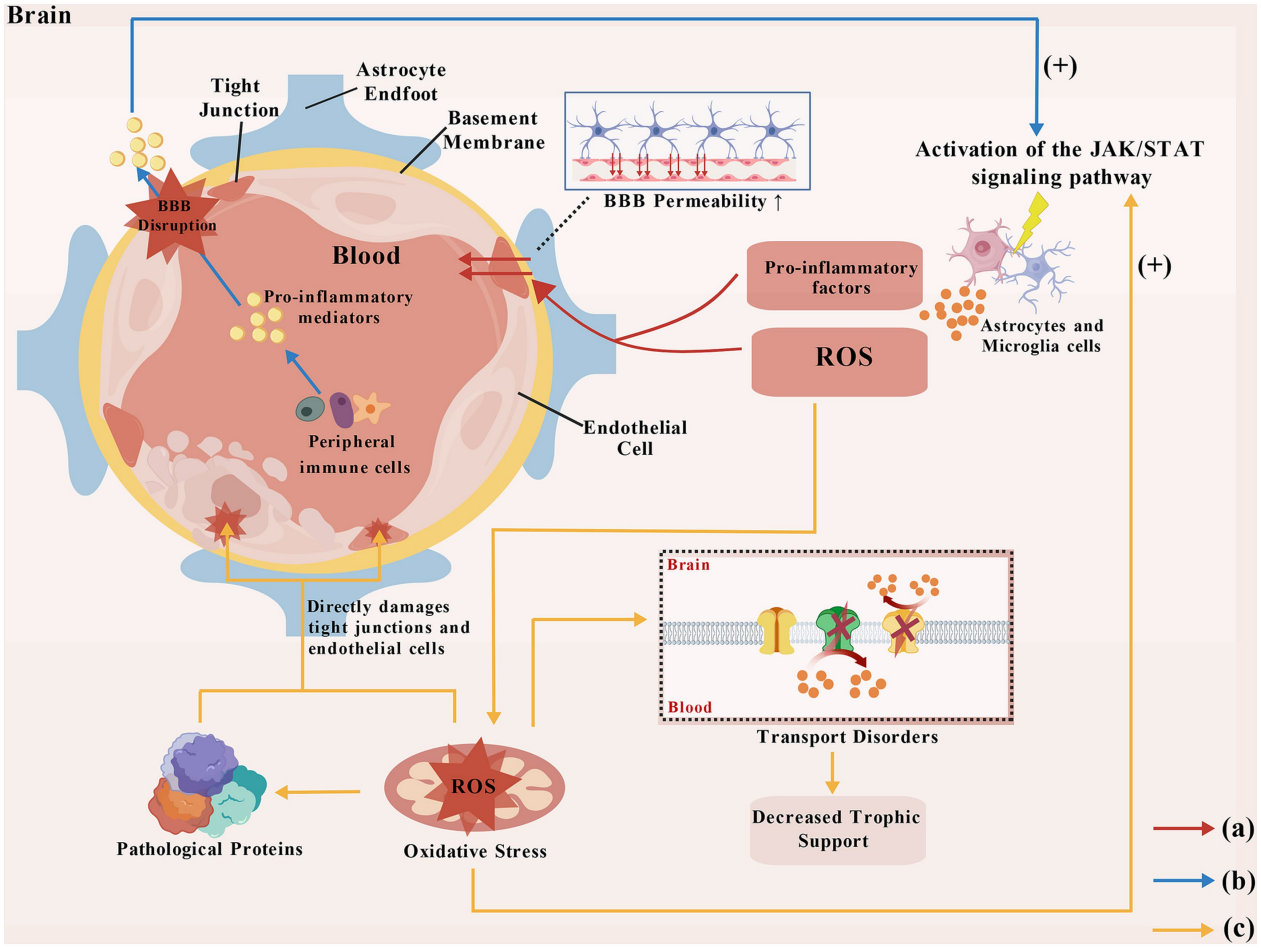
Cellular and molecular mechanisms of BBB disruption. This illustration depicts the pathological process by which peripheral immune signals interact with central nervous system cells to disrupt BBB integrity. Activated peripheral immune cells, astrocytes, and microglia release pro-inflammatory factors and ROS that disrupt the BBB through three interconnected pathways: Red arrow (a) Intracellular signaling activation and direct structural damage: Pro-inflammatory factors activate the JAK/STAT pathway in endothelial and glial cells, inducing massive production of inflammatory mediators and ROS that directly damage tight junction proteins and endothelial structure, thereby increasing BBB permeability. Blue arrow (b) Peripheral-central inflammatory cascade amplification: Peripheral immune cells and inflammatory mediators infiltrate brain tissue through the compromised BBB, activating local JAK/STAT signaling and establishing a positive feedback loop that sustains neuroinflammation. Yellow arrow (c) Oxidative stress-mediated dysfunction and impaired nutritional support: ROS accumulation triggers oxidative stress, causing oxidative damage to key functional proteins and disruption of trans-barrier transport mechanisms, ultimately leading to impaired clearance of metabolic waste, reduced neurotrophic factor supply, and neuronal homeostasis imbalance. These interconnected pathways collectively increase BBB permeability and drive the progression of neuroinflammation and related disorders (created with BioGDP.com; Jiang et al., 2025).

In this context, under conditions of neuroinflammation, activated CNS-resident glial cells can disrupt BBB endothelial function by secreting reactive oxygen species (ROS) (Chen T. et al., 2024; Pun et al., 2009). Furthermore, once BBB integrity is compromised, peripheral inflammatory cells and molecules can enter the CNS, triggering neuroinflammation and neuronal damage (Wu et al., 2021; Kalra et al., 2022) (Figure 2). Studies have shown that activation of the JAK/STAT pathway contributes to BBB dysfunction. In cerebral malaria models, components of Plasmodium falciparum can activate the JAK/STAT pathway, driving microglial polarization, disrupting the BBB, increasing microvascular permeability, and inducing ferroptosis and a type I IFN response. The JAK1/2 inhibitor ruxolitinib has been shown to effectively alleviate these pathological changes and protect BBB integrity (Piatti et al., 2025), similarly, the JAK inhibitor baricitinib has also been observed to exert a protective effect on the BBB (Matsushita et al., 2023; Chen et al., 2020; Kim J. et al., 2021). However, the JAK/STAT pathway is also considered to play a crucial role in neuroprotection; for example, erythropoietin-mediated JAK2/STAT3 activation can upregulate Bcl-2/Bcl-xL expression, thereby inhibiting apoptosis (Zhao et al., 2011), demonstrating neuroprotective functions.

### 2.2 The JAK/STAT pathway mediates neuroinflammation and pathological changes induced by glial cells

#### 2.2.1 Microglia

Microglia are central immune cells. In NDDs such as AD (Leng and Edison, 2021; Malpetti et al., 2020; Johnson et al., 2020), PD (Tansey et al., 2022; Smajić et al., 2022), HD (Rocha et al., 2021; Wilton and Stevens, 2020), and ALS (Humphrey et al., 2023; Dols-Icardo et al., 2020; Beers and Appel, 2019; Tam et al., 2019), microglial activation is considered a common pathological feature, manifested by changes in microglial numbers and morphology, elevated cytokine levels, oxidative stress, and progressive neuronal loss. Furthermore, studies have found that microglial activation is associated with various stimuli, including lipopolysaccharide (LPS), pesticides, misfolded *β*-amyloid and *α*-synuclein, and even air pollutants (Lull and Block, 2010; Fernando and Wijayasinghe, 2021). Some studies have reported that JAK/STAT activation in microglia serves as a chronic source of cytokines and ROS, thereby driving neuroinflammation and leading to progressive neuronal damage, which may be significantly linked to the chronic nature of NDDs (Block and Hong, 2007). Both LPS and IFN-*γ* are important factors that induce microglia to cause neuroinflammation. Specifically, LPS can overexpress TLR4, which then triggers NF-κB, AP-1, STAT5, interferon regulatory factors (IRFs), and other pro-inflammatory transcription factors via the TRIF and MyD88 pathways (Platanitis and Decker, 2018; Goulopoulou et al., 2016). In contrast, IFN-γ binds to IFN-γ receptors 1 and 2 (IFN-γR1/2) and activates the JAK/STAT cascade, thereby phosphorylating STAT1 and other IRFs and facilitating their translocation into the cell nucleus (Ivashkiv, 2018). Furthermore, high expression of MHC-II and the CD86 in microglia (Boche et al., 2013; Chhor et al., 2013) increases the production of pro-inflammatory mediators such as IL-6 and TNF-α, thereby promoting the activation and recruitment of microglia, macrophages, and CD4 T cells, which in turn exacerbates neuroinflammation, oxidative stress, and the loss of dopaminergic neurons (Yan et al., 2018).

#### 2.2.2 Astrocytes

Astrocytes are the most abundant type of glial cells in the CNS. When activated, they are referred to as reactive astrocytes, which are a hallmark pathological feature of NDDs and play a crucial role in neuroinflammation and neurodegeneration (Patani et al., 2023). STAT3 is primarily localized in astrocytes in the brain and plays a crucial role in inducing the transition of astrocytes from a quiescent to a reactive state (Kang and Hébert, 2011; Choi et al., 2020). Furthermore, the specific activation of STAT3 in reactive astrocytes has been confirmed in multiple studies involving animal models of NDDs (Ben Haim et al., 2015; Shibata et al., 2010) and human patients (Lu et al., 2013).

##### 2.2.2.1 Promotion of neuroinflammation and increase of Aβ production in AD

It is currently believed that the misfolding of Aβ and the abnormal accumulation of tau protein are both key factors in the neurodegenerative process of AD, and that Aβ accumulation can also trigger or accelerate the progression of tau pathology (Karran et al., 2011). It has been reported that pathological factors such as Aβ oligomers can induce reactive astrocyte proliferation by activating STAT3 (Choi et al., 2020; Olabarria et al., 2010), whereas specific knockout of STAT3 in astrocytes can alleviate neuroinflammation and damage, and improve brain network dysregulation in AD model mice (Reichenbach et al., 2019). Studies have found that reactive astrocytes may impair neural function through multiple pathways. On the one hand, they may generate excessive amounts of *γ*-aminobutyric acid via monoamine oxidase-mediated production and release through the BEST1 channel, thereby excessively inhibiting synaptic transmission and impairing synaptic function and cognitive ability (Jo et al., 2014); on the other hand, activated STAT3 promotes the release of pro-inflammatory cytokines, exacerbating the neuroinflammatory environment (Reichenbach et al., 2019); furthermore, it may also promote Aβ production by upregulating BACE1 expression, leading to the development of a vicious cycle. These changes collectively result in synaptic dysfunction and cognitive deficits (Choi et al., 2020). Therefore, inhibiting JAK/STAT3 phosphorylation, reducing Aβ production, and preventing astrocyte activation may represent a novel therapeutic strategy for early intervention in AD.

##### 2.2.2.2 Promotion of protein homeostasis and microglia activation in HD

The cytokine signaling suppressor 3 (SOCS3) is an important intracellular negative feedback regulator (Xiao et al., 2022). Studies have shown that selective overexpression of SOCS3 in astrocytes effectively inhibits JAK/STAT3 pathway activity, not only significantly reducing the abnormal activation of astrocytes and microglia but also decreasing the formation and size of mutant Huntington protein (mHTT) aggregates in neurons, accompanied by alleviation of striatal atrophy and restoration of glutamate homeostasis (Ben Haim et al., 2015; Abjean et al., 2023). This protective effect may be related to the high proteolytic capacity of astrocytes; after inhibiting their abnormal activation, this function is maintained or even enhanced.

However, activation of the JAK/STAT3 pathway may also exert a protective effect by enhancing the ability of neurons to clear mHTT and the clearance capacity of reactive astrocytes for mHTT (Abjean et al., 2023). Specifically, on the one hand, this pathway directly upregulates the expression of genes related to lysosomal biogenesis and various cathepsins, while simultaneously promoting the transcription of autophagy-related genes, thereby enhancing astrocytes’ ability to clear abnormal proteins via the autophagy-lysosomal pathway; on the other hand, STAT3 dimers can bind to the promoter regions of proteasome subunit genes, enhancing proteasome biosynthesis and assembly, and improving the degradation efficiency of the ubiquitin-proteasome system (Tydlacka et al., 2008), with these actions collectively contributing to the maintenance of protein homeostasis. Regarding the clearance of mHTT, studies have also shown that following activation of the JAK2-STAT3 pathway, dnaJ (a co-chaperone belonging to the heat shock protein family), produced by reactive astrocytes, participates in coordinating the proper folding and targeted degradation of mHTT (Abjean et al., 2023), reflecting the important protective role of the astrocyte heat shock response.

In addition, reactive astrocytes may also participate in the regulation of microglial activity through STAT3-dependent mechanisms, contributing to the positive feedback loop of neuroinflammation. Studies have shown that in HD mouse models, specific inhibition of the JAK/STAT3 pathway in astrocytes by SOCS3 reduces the mRNA levels of reactive microglial markers Iba1 and CD11b (Ben Haim et al., 2015). Furthermore, specific knockout of STAT in astrocytes has been shown to reduce hypoxia-ischemia-induced microglial reactivity (Hristova et al., 2016), specifically through the release of relevant factors by reactive astrocytes, which in turn reduces the production of transforming growth factor *β*-1 (TGF-β1) in microglia (Nobuta et al., 2012).

### 2.3 The JAK/STAT pathway mediates abnormalities in brain energy metabolism

The brain is the organ with the highest energy demand in the human body (Castrillon et al., 2023); however, another core pathological feature of NDDs is impaired brain energy metabolism (Cunnane et al., 2020). The JAK/STAT pathway is known to be involved in energy regulation; it not only plays a crucial role in the signal transduction of various energy metabolism-related hormones and cytokines (Dodington et al., 2018), but also mediates the key role of leptin signaling in regulating brain energy metabolism. Specifically, after leptin binds to its receptor in the CNS, it activates the receptor and recruits JAK2, leading to the phosphorylation of tyrosine residues in its intracellular domain (Hekerman et al., 2005), thereby regulating energy homeostasis and immune function (Myers et al., 2021; Pérez-Pérez et al., 2017). STAT3 is a key substrate for leptin signaling, activated by JAK protein through phosphorylation; the specific mechanism is as follows: upon activation, the leptin receptor recruits JAK2 and phosphorylates its intracellular domains (e.g., Tyr1138) (Kloek et al., 2002), thereby providing an anchoring site for STAT3 and activating the JAK2/STAT3 signaling pathway (Casado et al., 2023), which is closely associated with pathological processes such as aging, neuroinflammation, and NDDs (Patani et al., 2023; Liu and Sabatini, 2020).

Studies have also shown that weight loss, an emerging hallmark of AD, leads to a decrease in circulating leptin levels and may be a significant factor in the onset of dementia (Lieb et al., 2009). Furthermore, long-term leptin intervention has been found to significantly improve cognitive function in transgenic animal models (Tezapsidis et al., 2009), where leptin treatment not only helps reduce AMPK pathway-mediated tau phosphorylation (Salminen et al., 2011; Greco et al., 2008) but also increases the uptake of apolipoprotein E-dependent *β*-amyloid in cells and reduces the concentration of extracellular β-amyloid in the brain (Fewlass et al., 2004). In summary, leptin plays a crucial role in reversing the pathology of NDDs through its interactions with its receptors and downstream signaling pathways (Procaccini et al., 2016).

### 2.4 The JAK/STAT signaling pathway mediates neuroinflammation and contributes to demyelination in MS

The pathogenesis of MS is a complex process involving immunity, inflammation, and neurodegeneration. It begins with the disruption of peripheral immune tolerance, followed by a reduction in regulatory T cell function or the abnormal activation of effector B/T cells, which triggers an autoimmune response, leading to significant BBB dysfunction and the formation of active demyelinating lesions (Sun et al., 2024). Among these, T helper (Th) cells, such as the Th1 and Th17 subsets, play a central role: Th1 cells produce IFN-*γ*, which is closely associated with disease severity; Th17 cells release IL-17. It is precisely these cytokines, together with IL-12, IL-23, and others, that create a strong pro-inflammatory environment in the CNS (Naserpour et al., 2025).

During inflammatory injury, cytokines such as IL-6, IL-12, and IFN-γ, along with the abnormal differentiation of immune cells they mediate, contribute to the pathogenesis of demyelination. The JAK/STAT signaling pathway serves as a key hub, linking cytokine signaling to Th cell responses, and collectively drives the progression of myelin damage (Günaydın et al., 2021). Specifically, IL-12 drives Th1 cell differentiation by activating JAK2/TYK2 and downstream STAT4; IL-6 and IL-23, in turn, promote Th17 cell differentiation via the JAK1/2/STAT3 pathway (Naserpour et al., 2025). Notably, there is also complex cross-regulation among STAT proteins: activated STAT3 not only antagonizes NF-κB signaling but also induces tolerogenic dendritic cells to secrete anti-inflammatory factors such as IL-10 and TGF-β; meanwhile, STAT5a upregulates SOCS3 expression in CD4^+^ T cells, thereby negatively regulating IL-12-mediated Th1 differentiation (Hatami et al., 2018). Furthermore, IL-12-activated STAT4 promotes IFN-γ expression, which in turn enhances the transcription of IFN-regulated genes in macrophages via the JAK1/2/STAT1 pathway, forming a positive feedback loop that exacerbates the inflammatory response (Morandi et al., 2024).

**Figure 3:**
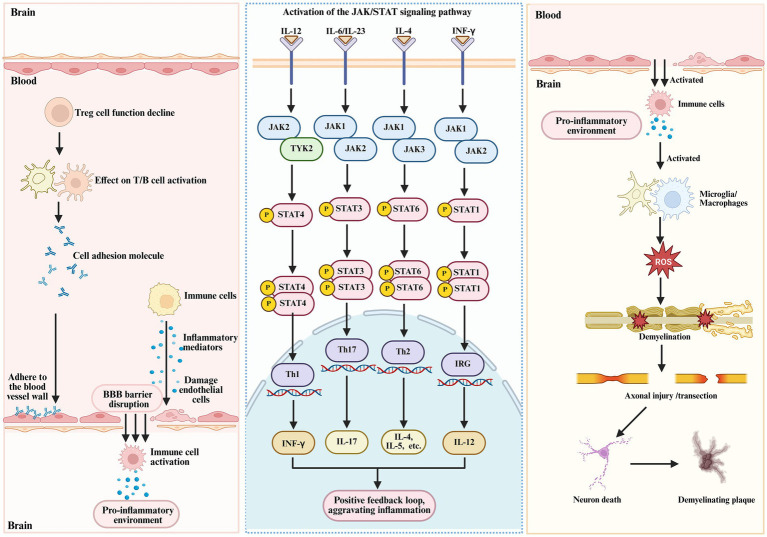
Schematic diagram of the pathogenesis of multiple sclerosis. This figure illustrates the core processes in MS development, from peripheral immune activation to central nervous system neurodegeneration. The process is initiated by peripheral immune activation and infiltration, characterized by dysfunctional regulatory T cells and aberrant activation of effector T/B cells. These cells secrete adhesion molecules that bind to CNS vascular walls, followed by the release of inflammatory cytokines that damage the BBB, enabling immune cell entry into the CNS. Subsequently, the JAK/STAT signaling pathway mediates inflammatory amplification, where cytokines activate specific JAK/STAT signaling axes to drive pathogenic T-cell subset differentiation (including Th1 and Th17), while IFN-γ establishes a positive feedback loop through the JAK1/2/STAT1 pathway that intensifies inflammation. Ultimately, central nervous system injury occurs as activated immune cells and microglia/macrophages create a pro-inflammatory environment, directly causing demyelination, axonal damage, and neuronal death, resulting in demyelinating plaques and irreversible neurological deficits (by BioRender).

Persistent inflammatory responses lead to increased microglial activation, macrophage infiltration, and an increase in the number of atypical CD8 T cells, which in turn trigger oligodendrocyte damage and myelin degradation. The loss of myelin leaves axons vulnerable to attack, ultimately and inevitably leading to axonal damage and neuronal death. Pathologically, this manifests as active demyelinating plaques and significant thinning of the frontal and temporal cortices in patients, reflecting an overall pathological progression from acute inflammation to chronic neurodegeneration (Sun et al., 2024; Naserpour et al., 2025) (Figure 3).

## 3 Small-molecule JAK/STAT inhibitors

Currently, JAK/STAT-related inhibitors have demonstrated broad application potential in the treatment of inflammatory diseases, autoimmune diseases, and cancer (Kubo et al., 2023; Chovatiya and Paller, 2021). Furthermore, several JAK inhibitors (such as ruxolitinib, tofacitinib, and baricitinib) have already been approved by the U.S. Food and Drug Administration (FDA) for the treatment of autoimmune diseases and certain types of cancer (Saharinen et al., 2000). However, existing research on the JAK/STAT pathway in the field of NDDs is fragmented and lacks systematic organization. Therefore, we conducted a systematic search of the PubMed database over the past decade for relevant literature (“Alzheimer’s Disease” OR “Parkinson’s Disease” OR “Huntington’s Disease” OR “Amyotrophic Lateral Sclerosis” OR “Multiple Sclerosis”) AND (“JAK/STAT Signaling Pathway”) and clinical trials on the ClinicalTrials.gov platform. We compiled a list of various NDDs-related drugs and discussed their mechanisms of action and therapeutic efficacy.

### 3.1 JAK/STAT inhibitors in AD

#### 3.1.1 Baricitinib

Baricitinib is an oral JAK1/JAK2 selective inhibitor that exerts anti-inflammatory effects by downregulating the transcription of pro-inflammatory cytokines. In recent years, this drug has been approved by the FDA for the treatment of autoimmune diseases such as rheumatoid arthritis (RA) (Urits et al., 2020; Mogul et al., 2019) and alopecia areata (Freitas et al., 2023; Singh and Driscoll, 2023). In an ovarianectomy/D-galactose (OVX/D-gal)-induced AD rat model, baricitinib treatment effectively inhibited JAK2 and STAT3 phosphorylation, reduced levels of pro-inflammatory cytokines and oxidative stress markers, and alleviated neuroinflammation; simultaneously, the drug can reverse histopathological changes, reduce reactive astrocyte proliferation and Aβ plaque deposition, thereby preventing neuronal death and synaptic dysfunction (Hindam et al., 2024).

#### 3.1.2 Tofacitinib (CP-690,550)

Tofacitinib is a JAK inhibitor that preferentially inhibits the JAK1 and JAK3 subtypes, while also inhibiting JAK2. It exerts anti-inflammatory effects by blocking the activation of the NLRP inflammasome in neutrophils and the production of IL-1β (Montoya et al., 2018). Currently, this drug has been approved by the FDA for the treatment of inflammatory autoimmune diseases (Roskoski, 2024), such as RA (Dhillon, 2017; Rakieh and Conaghan, 2013), psoriatic arthritis (Berekmeri et al., 2018), and ulcerative colitis (Anirvan and Giri, 2024; Singh et al., 2024; Lair-Mehiri et al., 2020; Steenholdt et al., 2023). In an AD rat model, following activation of the NLRP3 inflammasome via intraperitoneal injection of LPS, tofacitinib treatment inhibited JAK signaling, further improved spatial memory deficits caused by NLRP1 inflammasome activation, and reduced pyroptosis-related inflammation levels (Yang et al., 2021).

#### 3.1.3 Vildagliptin

Vildagliptin is a dipeptidyl peptidase-4 inhibitor commonly used in clinical practice to treat type 2 diabetes (Keating, 2010). By inhibiting DPP-4 activity, it increases levels of glucagon-like peptide-1 and glucose-dependent insulinotropic polypeptide, thereby enhancing insulin secretion and suppressing glucagon release, thus exerting its hypoglycemic effect (Henness and Keam, 2006). In an AlCl_3_-induced AD rat model, vildagliptin treatment activated the AKT/ERK1/2 signaling pathway, promoted its phosphorylation, significantly reduced *STAT3* mRNA expression and JAK2 phosphorylation levels in the hippocampus and simultaneously reversed the downregulation of *Klotho* mRNA (Yossef et al., 2020), thereby exerting neuroprotective, anti-apoptotic, and anti-inflammatory effects.

#### 3.1.4 TTP488 (Azeliragon)

TTP488 is an orally active, centrally acting receptor of advanced glycation end products antagonist that is clinically used to inhibit tumor proliferation and metastasis and has a favorable safety profile (Alka et al., 2024; Magna et al., 2023). It is currently in Phase III clinical trials. In an 18-month randomized, placebo-controlled trial involving 399 patients, the low-dose TTP488 group (initial 15 mg/day, subsequently 5 mg/day) had ADAS-cog (AD Assessment Scale-Cognitive Subscale) scores that were 2.7 points lower than those in the placebo group (*p* = 0.03), indicating a statistically significant trend toward cognitive improvement (Burstein et al., 2014). Concurrently, preclinical studies have shown that in animal models of AD, TTP488 reduces A*β* plaque deposition, regulates amyloid precursor protein metabolism, decreases neuroinflammatory responses, and improves cerebral blood flow and cognitive function (Burstein et al., 2018). Further studies found that TTP488 can improve spatial memory in AD model rats, reduce NLRP1 expression and neuronal damage, and inhibit caspase-1 and IL-1β activity. Research has confirmed that its anti-inflammatory effects are related to the inhibition of NLRP1 inflammasome activation (Yang et al., 2021), and this anti-inflammatory function may be associated with the regulation of the JAK/STAT signaling pathway. *In vitro* experiments using AD cell models revealed that Aβ-induced SH-SY5Y cells treated with TTP488 exhibited reduced expression of inflammatory factors and restored cell viability. Furthermore, the study found that TTP488 inhibits the upregulation of NLRP3 by blocking the activation of the JAK1/STAT3/NF-κB/IRF3 pathway (Xue et al., 2021).

These findings suggest that TTP488 may play a crucial role in the treatment of AD by inhibiting the JAK/STAT signaling pathway and blocking inflammasome activation, and it holds promise as a potential therapeutic agent for slowing the progression of mild AD.

#### 3.1.5 SPA1413

SPA1413 is an isoflavone derivative derived from S-estrogene, a metabolite of gut bacteria, which inhibits β-amyloid aggregation, alleviates cytotoxicity and neuroinflammatory responses, and exerts neuroprotective effects (An et al., 2022). *In vitro* experiments have shown that in an LPS-stimulated BV2 microglial cell model, SPA1413 significantly inhibits the expression of pro-inflammatory mediators such as IL-6 and TNF-*α*, and reduces IL-11 levels by inhibiting the JAK/STAT signaling pathway; simultaneously, the compound also attenuates neuron death mediated by activated microglia (Yoon et al., 2023). In summary, SPA1413 exerts anti-neuroinflammatory and neuroprotective effects by inhibiting the JAK/STAT signaling pathway and may represent a potential candidate drug with therapeutic value for AD.

#### 3.1.6 Hydroxychloroquine (HCQ)

HCQ is a chloroquine derivative with both antimalarial and anti-inflammatory properties. It is widely used clinically to treat malaria (Zhou et al., 2020), systemic lupus erythematosus (Nestor et al., 2024; Shipman et al., 2020), and RA (Quiñones et al., 2023).

In a study based on large-scale epidemiological data, among patients with RA, the use of HCQ was associated with an 8–16% reduced risk of AD and related dementias in older adults compared to methotrexate; this protective effect was dose-dependent and increased with prolonged treatment duration (Varma et al., 2023). At the mechanistic level, HCQ exhibits multiple neuroprotective effects in *APP/PS1* transgenic AD mouse models (Varma et al., 2023); it significantly inhibits STAT3 phosphorylation in the hippocampus, thereby blocking the JAK/STAT3 signaling pathway in microglia and astrocytes, suppressing neuroinflammation, promoting Aβ clearance by microglia, and reducing tau protein phosphorylation. These effects may be closely related to HCQ-mediated inactivation of the STAT3 signaling pathway, which plays a critical role in various pathological processes of AD.

#### 3.1.7 Stattic

Stattic is a small-molecule inhibitor that blocks STAT3 activation and dimerization; it is not only a non-peptide small molecule that selectively inhibits the function of the STAT3 SH2 domain, but also selectively inhibits STAT3 activation and nuclear translocation, and induces apoptosis in STAT3-dependent cancer cell lines (Schust et al., 2006).

A recent study using *in vitro* and *in vivo* experiments, conducted on 5XFAD mice—a model of AD—and primary astrocytes treated with oligomeric Aβ, found that in the early stages of AD, there is a significant increase in reactive astrocytes in the dentate gyrus of the hippocampus, and their activation is closely associated with elevated levels of STAT3 phosphorylation (Choi et al., 2020). By inhibiting STAT3 phosphorylation, Stattic not only reversed the abnormal activation of astrocytes but also reduced BACE1 expression and Aβ production in 5XFAD mice, thereby significantly improving learning and memory impairments in the model mice (Choi et al., 2020). These results indicate that the STAT3 inhibitor Stattic can induce activated astrocytes to transition to a quiescent state, potentially providing a novel strategy for AD treatment.

#### 3.1.8 Fludarabine

Fludarabine is a chemotherapy drug used to treat various hematologic malignancies. Its mechanism of action involves inhibiting STAT1 phosphorylation, thereby blocking STAT1-mediated signaling pathways at downstream levels; however, this effect occurs in both normal and cancer cells (Plosker and Figgitt, 2003; Feng et al., 2017).

In vitro and in vivo studies have shown that, in C57BL/6 mice and RAW264.7 cells exposed to LPS, fludarabine significantly reduced STAT1/IRF1 protein expression, inhibited neutrophil and macrophage infiltration, and decreased the release of NO, TNF-*α*, and IFN-*γ*, indicating that fludarabine possesses anti-inflammatory effects (Lee et al., 2025) (Table 1).

**Table 1:** Research on JAK/STAT pathway-related inhibitors in AD.

Drug	Experimental model	Target	Effect	References
Baricitinib	Ovariectomized/D-galactose-treated rat model	JAK1/2	Significantly reduced expression levels of p-JAK2 and p-STAT3, and alleviated neuroinflammation.	Hindam et al. (2024)
	Physiologically based pharmacokinetic model	JAK1/2	The low brain permeability of baricitinib limits its repurposing for Alzheimer’s disease.	Faquetti et al. (2024)
Tofacitinib	LPS-induced AD rat model	JAK1, JAK2 and JAK3	Improve spatial memory and reduce inflammatory levels associated with pyroptosis.	Yang et al. (2021)
Hydroxychloroquine	Active control, observational cohort study	JAK/STAT3	Reduce the risk of developing ADRD and protect against AD-related anomalies.	Varma et al. (2023)
	*APP/PS1* transgenic mouse model			
Stattic	5XFAD mouse model	STAT3	Reverse the activation state of astrocytes to a resting state.	Choi et al. (2020)
Fludarabine	LPS-induced C57BL/6 mouse and RAW264.7 cell model	STAT1	Reduce cell pyroptosis-related inflammation levels.	Lee et al. (2025)
Vildagliptin	Drug-induced metabolic syndrome rat model of AD	JAK2/STAT3	Reduce inflammatory, apoptotic, and oxidative stress biomarkers in the hippocampus.	Yossef et al. (2020)
SPA1413	LPS-stimulated BV2 Cell model	JAK/STAT	Reduce LPS-activated microglia to inhibit neuronal cell death.	Yoon et al. (2023)
TTP488	Aβ-induced AD SH-SY5Y cell model	JAK1/STAT3	Reduce cell apoptosis and expression of inflammatory factors.	Xue et al. (2021)
	Aβ-induced AD SH-SY5Y cell model	JAK1/STAT3	Promote cell proliferation and reduce cell apoptosis, and expression of inflammatory factors.	Yang et al. (2021)

### 3.2 JAK/STAT inhibitors in PD

#### 3.2.1 AZD1480

AZD1480 is an orally active, competitive JAK1/2 small-molecule inhibitor primarily used to inhibit tumor growth (Murakami et al., 2014; Maenhout et al., 2014); however, due to its good tolerability and lack of immunosuppressive effects, it is also considered a potential therapeutic option for PD.

In an in vitro experiment, AZD1480 effectively inhibited STAT1/STAT3 phosphorylation in primary mouse microglia and macrophages induced by α-synuclein (α-SYN), and reduced the expression of MHC-II molecules and inflammatory gene levels (Qin et al., 2016). Concurrently, in vivo experiments revealed that in a rat model with viral overexpression of α-SYN, treatment with AZD1480 significantly inhibited microglial activation, macrophage and CD4 + T cell infiltration, and the production of pro-inflammatory cytokines, thereby alleviating α-SYN-induced neuroinflammation and reversing the abnormal expression of inflammation-related genes in the substantia nigra (Qin et al., 2016). Another study utilized α-SYN preformed fibrils (PFFs) injected into Line 61 transgenic mice to establish a novel PD acceleration model (the Line 61-PFF mouse model), and found that inhibition of the JAK/STAT pathway significantly reduced neuroinflammation (Hong et al., 2024). By blocking this pathway, AZD1480 not only reduced α-SYN pathology and MHC class II molecule expression but also inhibited the activation and aggregation of microglia, macrophages, and T cells (Hong et al., 2024). Single-cell transcriptomic analysis also revealed that JAK inhibition specifically downregulates the expression of antigen-presenting-related genes in pathogenic monocyte/macrophage clusters, as well as cytokine and complement genes in pro-inflammatory T-cell clusters (Hong et al., 2024). These findings collectively indicate that the JAK/STAT pathway suppresses neuroinflammation and neurodegeneration by regulating innate and adaptive immune responses, and that targeting this pathway represents a viable strategy for neuroprotective therapy in NDDs.

#### 3.2.2 Apamin (APM)

APM is a potent, selective antagonist (Moreno and Giralt, 2015) of small-conductance calcium-activated potassium channels (SK channels) identified in apitoxin, exhibiting multiple pharmacological activities including anticancer, anti-arthritic, and neuroprotective effects (Aufschnaiter et al., 2020). It may also play a role in suppressing neuroinflammation in NDDs (Kim H. et al., 2021).

Recent studies have shown that in LPS-stimulated BV2 cells and primary rat microglia, APM effectively inhibits the nuclear translocation of NF-κB and STAT3, as well as the activation of the MAPK/ERK signaling pathway. Since STAT3 acts as a key inflammatory transcription factor, its inhibition significantly reduces the release of pro-inflammatory cytokines and the abnormal activation of microglia (Park et al., 2020). Furthermore, in 1-methyl-4-phenylpyridine (MPP^+^)-induced SH-SY5Y cells and rat primary dopaminergic neuron models, APM was found to inhibit STAT3 phosphorylation, MAPK/ERK nuclear translocation and DNA-binding activity, and to alleviate MPP^+^-induced Ca^2+^ overload, thereby significantly reducing damage and loss of dopaminergic neurons (Park et al., 2022).

In summary, APM is considered a potent inhibitor of neuroinflammation. By regulating SK channels in microglia and synergistically inhibiting multiple signaling pathways, including MAPK/ERK, NF-κB, and STAT3, it exerts potent anti-neuroinflammatory and neuroprotective effects.

#### 3.2.3 LM-021 and NC009-1

The coumarin-chalcone derivative (LM-021) (Chiu et al., 2021) and the indole derivative (NC009-1) (Chang et al., 2017) are two compounds developed by Yang et al. Studies have shown that these compounds can inhibit inflammatory responses and oxidative stress, reduce ROS production, and promote neurite outgrowth, exerting neuroprotective effects by regulating relevant signaling pathways (Chi et al., 2025). Specifically, NC009-1 improves motor and non-motor symptoms and increases striatal dopamine and dopamine transporter levels (Chiu et al., 2023), while LM-021 exerts anti-neuroinflammatory activity by specifically inhibiting the JAK2/STAT3 signaling pathway (Chen I. C. et al., 2024). These findings suggest that both compounds may be involved in regulating the pathological mechanisms of PD and have the potential to serve as therapeutic agents for PD. This protective effect was also confirmed in BV2 cells, where researchers found that NC009-1 and LM-021 can inhibit p-JAK2 and p-STAT3 levels and restore SOCS3 expression, thereby exerting anti-neuroinflammatory effects (Yang et al., 2023).

#### 3.2.4 Tofacitinib

Tofacitinib is a selective inhibitor of JAK1 and JAK3. In a mouse PD model induced by 1-methyl-4-phenyl-1,2,3,6-tetrahydropyridine, a study found that tofacitinib significantly improved behavioral deficits, neuroinflammatory responses, and dopaminergic neuron damage in model mice by inhibiting the JAK/STAT pathway (Alshammari et al., 2022), suggesting that JAK-targeted immunomodulatory strategies may offer new avenues for the treatment of PD.

#### 3.2.5 Irbesartan and flavonoids

Irbesartan is an angiotensin II type 1 receptor blocker primarily used clinically to treat hypertension; it also possesses antioxidant and anti-inflammatory properties (Mirghaed et al., 2025; Saad et al., 2021). Flavonoids, as angiotensin-converting enzyme 2 activators, can alleviate cerebral ischemia/reperfusion injury and promote brain tissue repair (Abdel-Fattah et al., 2018). Both compounds possess anti-inflammatory activity and may hold potential value in the treatment of neuroinflammatory diseases.

*In vitro* studies indicate that in a rotenone-induced PD model, the combined use of irbesartan and flavonoids can synergistically regulate the *miR-155*/SOCS1/STAT1/STAT6 signaling axis and phosphatase systems such as PP2A and MKP-1, inhibit STAT1 activation, and promote STAT6 phosphorylation. This, in turn, drives the polarization of microglia from the pro-inflammatory M1 phenotype to the anti-inflammatory M2 phenotype, ultimately exerting neuroprotective and anti-inflammatory effects (Alhadad et al., 2025). These findings suggest that these two drugs possess multi-target therapeutic potential for the intervention of NDDs (Table 2).

**Table 2:** Research on JAK/STAT pathway related inhibitors in PD.

Drug	Experimental model	Target	Effect	References
AZD1480	α-SYN-induced microglia and macrophages model	JAK1/2	Suppresses MHC class II molecule and inflammatory gene expression.	Hong et al. (2024)
	Viral overexpression α-SYN-induced PD rat model		Inhibit neuroinflammation.	
	α-SYN-induced mouse primary macrophage and microglial cell models	JAK1/2	Inhibit neuroinflammation and dopaminergic neurodegeneration.	Qin et al. (2016)
	Viral overexpression α-SYN-induced PD rat model			
Apamin	LPS-induced mouse microglial BV2 cell line and rat primary microglial models	STAT3	Significantly reduce the release of pro-inflammatory cytokines and abnormal activation of microglia.	Park et al. (2020)
	MPP^+^-induced SH-SY5Y human dopaminergic neuroblastoma cell line and rat primary mesencephalic dopaminergic neuron models	STAT3	Alleviate damage to dopaminergic neurons.	Park et al. (2022)
LM-021 and NC009-1	Mouse BV2 microglial cell model	JAK2/STAT3	Anti-neuroinflammatory effects	Yang et al. (2023)
Tofacitinib	Parkinson’s disease 1-methyl-4-phenyl-1,2,3,6-tetrahydropyridine (MPTP) model	JAK1, JAK2 and JAK3	Improve gross motor activity and reversed catatonia scores.	Alshammari et al. (2022)
Irbesartan and Flavone	PD rat model	JAK/STAT	Inhibit neuroinflammation through microglial polarization.	Alhadad et al. (2025)

### 3.3 JAK/STAT inhibitors in HD

#### 3.3.1 Cilostazol

Cilostazol is a selective phosphodiesterase III inhibitor (Tan et al., 2021), primarily used clinically for antiplatelet therapy in cerebrovascular diseases (Zheng et al., 2019). However, some studies have revealed that this drug may also exert a potential therapeutic effect in NDDs by modulating neuroinflammatory responses (Jung et al., 2010; El-Ezz et al., 2024; Hedya et al., 2018).

An *in vitro* study demonstrated that in a 3-nitropropionic acid-induced HD rat model, El-Abhar et al. found that cilostazol significantly alleviated 3-nitropropionic acid-induced neuroinflammation and neuronal damage by inhibiting the activation of the IL-6/JAK2/STAT3 pathway and enhancing the negative feedback regulation of SOCS3, thereby exhibiting neuroprotective effects in the HD model (El-Abhar et al., 2018).

### 3.4 JAK/STAT inhibitors in ALS

#### 3.4.1 Ruxolitinib

Ruxolitinib is an oral selective JAK1/JAK2 inhibitor commonly used in clinical practice to treat immune-related diseases such as vitiligo (Yuan et al., 2024), graft-versus-host disease (Zhang et al., 2022), and atopic dermatitis (Simpson et al., 2025). Studies have shown that in the RAW264.7 macrophage cell line and *C9orf72* knockout mouse models, STAT1 protein and p-STAT1 levels are significantly elevated. Following ruxolitinib intervention, the upregulation of STAT1-regulated inflammatory genes was significantly inhibited at the cellular level, splenomegaly and lymphadenopathy were markedly alleviated at the tissue level, and STAT1 protein expression in splenic tissue was reduced (Pang and Hu, 2023). These results indicate that ruxolitinib has potential for the treatment of ALS by targeting the JAK/STAT pathway.

#### 3.4.2 Tofacitinib

Tofacitinib is an oral small-molecule JAK inhibitor widely used in clinical practice to treat conditions such as ulcerative colitis (López-Sanromán et al., 2021), systemic lupus erythematosus (Hou et al., 2024), and RA (Mortezavi and Mysler, 2023). Studies have shown that by inhibiting the JAK/STAT signaling pathway, this drug significantly suppresses the activation of natural killer cells and the secretion of pro-inflammatory factors, and has demonstrated neuroprotective effects on ALS-associated neurons in both in vitro and *in vivo* models (Figueroa-Romero et al., 2022). These findings support tofacitinib as a potential therapeutic strategy for ALS, particularly for pathways involving natural killer cell-mediated neuroinflammation and neuronal damage.

### 3.5 JAK/STAT inhibitors in MS

#### 3.5.1 Deucravacitinib

Deucravacitinib is an oral TYK2-specific inhibitor approved in 2022. It exerts its therapeutic effect by targeting the TYK2 pseudokinase (JH2) domain and has demonstrated clinical efficacy in trials for moderate-to-severe psoriasis (Hoy, 2022). Although its application is currently focused primarily on psoriasis, research suggests that psoriasis is significantly associated with an increased risk of MS, and the two share similarities in genetic risk variants and inflammatory pathways (Egeberg et al., 2016; Islam et al., 2019).

In an intraperitoneal injection mouse model of EAE (experimental autoimmune encephalomyelitis), the therapeutic efficacy of deucravacitinib was comparable to that of certain nanopolymers, effectively reducing neuroinflammation and improving clinical outcomes, suggesting that the JAK/STAT pathway inhibition strategy represented by deucravacitinib is a promising direction for MS treatment (Sharma et al., 2024). Notably, compared to first-generation pan-JAK inhibitors, deucravacitinib may offer superior safety due to its unique mechanism of action.

#### 3.5.2 Natalizumab

Natalizumab is a humanized anti-*α*4 integrin monoclonal antibody that blocks the interaction between α4*β*1 and α4β7 integrins and their endothelial receptors by binding to the α4 subunit, thereby reducing CNS inflammation (Yednock et al., 1992). Two large-scale, open-label post-marketing studies, STRATA and TOP, have confirmed that this drug is highly effective in treating relapsing–remitting MS (O'Connor et al., 2014; Butzkueven et al., 2014). One study found that microRNA-20b (*miR-20b*) expression was significantly downregulated in treatment-naïve patients with relapsing–remitting MS, whereas following treatment with IFN-β or natalizumab, *miR-20b* levels returned to those observed in healthy controls, suggesting that the beneficial effects of both therapies may be partially mediated through the regulation of *miR-20b* (Jafari Harandi et al., 2024). Further bioinformatics analysis revealed that *miR-20b* targets are significantly enriched in the JAK/STAT signaling pathway, suggesting that the efficacy of IFN-β and natalizumab in relapsing–remitting MS may be mediated by restoring *miR-20b* expression to baseline levels.

#### 3.5.3 Tofacitinib

A case report documented a 48-year-old female patient with both relapsing–remitting MS and RA. After receiving low-dose tofacitinib (5 mg/day), the patient’s RA went into remission, and she was successfully weaned off corticosteroids. More importantly, during several years of follow-up, her MS remained stable, with no clinical or radiological worsening observed (Sakellariou et al., 2025).

In animal models of MS, tofacitinib improved cuprizone-induced impairments in motor coordination and muscle strength and reduced levels of IFN-*γ*, IL-6, IL-1β, and TNF-α, demonstrating anti-inflammatory effects (Günaydın et al., 2021); simultaneously, the drug can suppress the elevation of matrix metalloproteinases (MMP-9 and MMP-2) and mitigate the loss of myelin integrity, which may be related to the inhibition of STAT3 and STAT5 phosphorylation (Günaydın et al., 2021). Furthermore, Th17 cells participate in the pathogenesis of MS and EAE models by infiltrating the CNS and producing effector molecules that bind to resident glial cells. Among these, Th17 cell-driven transcriptional changes in astrocytes are partially mediated by JAK1-dependent mechanisms (Milne et al., 2024), suggesting that tofacitinib, which targets JAK1, may exert therapeutic effects in MS.

#### 3.5.4 Rifampin

Rifampin is a semi-synthetic broad-spectrum antibiotic derived from rifamycin B. Due to its lipophilic nature, it easily crosses the BBB and has been shown to exert neuroprotective effects in various CNS disorders (Spudich et al., 2006; Yulug et al., 2014; Bi et al., 2013).

A study using a female C57BL/6 mouse model of experimental autoimmune encephalomyelitis (EAE) found that pre-induction administration of rifampin effectively alleviated clinical symptoms in EAE-affected mice (Ma et al., 2016). Mechanistic studies indicate that rifampin treatment significantly downregulates serum levels of the pro-inflammatory cytokine IL-6 and inhibits STAT3 phosphorylation (Ma et al., 2016), thereby blocking the IL-6/JAK/STAT3 pathway’s role in driving the differentiation of naive CD4 + T cells into Th17 cells (O'Shea and Plenge, 2012). This, in turn, reduces Th17 cell differentiation and the production of its key effector molecule, IL-17A, ultimately curbing neuroinflammation and autoimmune attacks on the CNS.

#### 3.5.5 BD750

BD750 is a benzothiazole derivative that blocks T-cell proliferation by modulating the JAK3/STAT5 signaling pathway (Liu et al., 2013). *In vitro* studies have shown that treatment of dendritic cells (DCs) with BD750 specifically inhibits JAK3/STAT5 phosphorylation, thereby “reprogramming” DCs into tolerant dendritic cells (tolDCs). These tolDCs exhibit an immature phenotype, with significantly downregulated expression of costimulatory molecules (such as MHC class II) and pro-inflammatory cytokines (particularly IL-12), thereby effectively suppressing T-cell proliferation in response to allogeneic antigen stimulation and reducing Th1 and Th17 responses (Zhou et al., 2017).

In the EAE mouse model used in this study, BD750-induced tolDCs, generated by adoptive transfer of myelin-expressing oligodendrocyte glycoprotein-loaded cells, suppressed the onset and progression of EAE and reduced the degree of inflammatory infiltration and demyelination in spinal cord tissue. Furthermore, treatment with tolDCs loaded with antigenic peptides significantly reduced the frequency of Th1 and Th17 cells in the spleens of EAE mice (Zhou et al., 2017). These results indicate that BD750, as an inhibitor of the JAK3/STAT5 signaling pathway, opens up an innovative strategy for treating MS by targeting the JAK/STAT pathway through the induction of tolDC-mediated immune tolerance.

#### 3.5.6 Tyrphostin AG490

Tyrphostin AG490 is a JAK/STAT inhibitor that primarily exerts its effects by inhibiting JAK2 and JAK3, with approximately 4.3 times greater potency against JAK2 than against JAK3 (Gonçalves et al., 2010). In an EAE mouse model, treatment with AG490 significantly reduced disease incidence, delayed the onset of disease, alleviated disease severity, and reduced inflammatory cell infiltration and demyelination in the CNS (Zhao et al., 2017). Furthermore, staining of splenic germinal centers revealed that the fluorescence intensity in the AG490 group was significantly lower than that in the vehicle group, indicating that germinal center responses were suppressed. Subsequent studies focused on follicular helper T (Tfh) cells, compared with the vehicle group, AG490 treatment significantly reduced the proportion of Tfh cells in mice and simultaneously downregulated the expression of their key transcription factor, Bcl-6 (Zhao et al., 2017). Given that Bcl-6 is a central regulator of Tfh cell differentiation, these findings suggest that AG490 may act by inhibiting JAK2 and JAK3, thereby disrupting STAT3-mediated Bcl-6 expression and Tfh cell differentiation, and ultimately alleviating neuroinflammation and improving disease symptoms by modulating the humoral immune response (Table 3).

**Table 3:** Research on JAK/STAT pathway related inhibitors in MS.

Drug	Experimental model	Target	Effect	References
Deucravacitinib	Molecular dynamics model	JAK/STAT	Inhibits JH1 activation and suppresses ATP binding to the domain.	Bao Y. et al. (2024)
Natalizumab	Case–control study	JAK/STAT	Restore *miR-20b* expression to baseline levels.	Jafari Harandi et al. (2024)
Tofacitinib	Cuprizone-induced MS mouse model	JAK1, JAK2 and JAK3	Promotes remyelination and enhances myelin integrity.	Günaydın et al. (2021)
	Female patient with relapsing–remitting MS and rheumatoid arthritis	JAK1, JAK2 and JAK3	No clinical or radiographic deterioration.	Sakellariou et al. (2025)
Rifampin	Myelin oligodendrocyte glycoprotein peptide (MOG₃₅-₅₅) induced EAE mouse model	JAK/STAT3	Suppress pathogenic Th17 cell responses.	Ma et al. (2016)
BD750	EAE mouse model	JAK3/STAT5	Ameliorates pro-inflammatory T cell responses and EAE in mice.	Zhou et al. (2017)
AG490	EAE mouse model	JAK2/3	Suppresses inflammatory cells and demyelination.	Zhao et al. (2017)

## 4 Clinical safety issues and adverse reactions of JAK/STAT-targeting drugs

JAK/STAT-targeting drugs have demonstrated multifaceted therapeutic potential in various non-deformity-related diseases; however, their clinical application still requires rigorous safety validation. Long-term real-world data from patients with RA provide important insights in this regard.

In a safety analysis of tofacitinib spanning Phase I to IIIb/IV clinical trials and involving a total of 7,061 RA patients over a period of up to 9.5 years, the results showed that infections and infectious diseases were the most common adverse events during treatment (56.2%), specifically including viral upper respiratory tract infections (17.3%), other upper respiratory tract infections (17.2%), urinary tract infections (11.8%), and bronchitis (11.3%). Additionally, 26.3% of patients experienced serious adverse events, and 23.1% discontinued treatment due to adverse events; the primary causes of death were cardiovascular disease, infections, and respiratory diseases. Further analysis identified tofacitinib dose, advanced age, and Asian ethnicity as independent risk factors for infectious events (Cohen et al., 2020).

Similar trends were also observed in the long-term safety assessment of baricitinib. This study, which enrolled 3,770 patients with moderate-to-severe active RA, was a 9.3-year safety evaluation of baricitinib based on 9 randomized clinical trials and long-term extension studies. The results showed that infections remained the most common adverse events, with severe infections primarily consisting of pneumonia, herpes zoster, urinary tract infections, and cellulitis. In addition, the study reported malignancies of respiratory and mediastinal origin, as well as those of the breast and gastrointestinal tract, along with 23 cases of diverticulitis and 7 cases of lower gastrointestinal perforation. Notably, however, the incidence of these adverse events did not show a cumulative increase with prolonged treatment duration (Taylor et al., 2022).

An analysis of the World Health Organization database further revealed the adverse event profiles of different JAK inhibitors. In long-term treatment reports, ruxolitinib, tofacitinib, and baricitinib ranked in the top three in terms of the number of adverse event reports (Hoisnard et al., 2022); disproportionality analysis estimated that herpesvirus infections had the highest probability of occurrence (IC025 = 1.7), followed by musculoskeletal and connective tissue disorders (IC025 = 1.1), and then benign, malignant, and unspecified neoplasms (IC025 = 0.8). Notably, the increased incidence of infection-related adverse events was significantly correlated with the dosage of baricitinib or tofacitinib, whereas the overreporting of tumors was unrelated to the dosage of either agent (Hoisnard et al., 2022).

In summary, JAK inhibitors, represented by tofacitinib and baricitinib, pose risks of infection, serious adverse events, and potential tumor-related risks during long-term use. Although the incidence of some adverse events did not increase with cumulative exposure over time, advanced age, high-dose therapy, and geographic region may all be risk factors for infectious events. Therefore, when expanding the use of JAK/STAT inhibitors to elderly NDD patients requiring long-term treatment, the therapeutic benefits must be carefully weighed against safety risks such as infections and neoplasms, and prospective pharmacokinetic and safety studies targeting this specific population must be conducted.

## 5 Future prospects

### 5.1 Major JAK/STAT inhibitors: structure–activity relationships and their impact on the treatment of neurodegenerative diseases

As the central role of the JAK/STAT signaling pathway in the pathological progression of NDDs becomes increasingly clear, the development of JAK/STAT inhibitors with CNS activity has become a focus of drug discovery. However, existing JAK inhibitors were mostly designed for peripheral immune disorders, and their application in NDDs is limited by poor BBB permeability, insufficient selectivity, and long-term safety concerns. Therefore, a thorough understanding of the structure–activity relationships of these inhibitors and clarification of how their structural features influence selectivity, BBB penetration, pharmacokinetic properties, and therapeutic efficacy in NDDs are of great significance for the rational design of next-generation CNS-targeted inhibitors.

#### 5.1.1 General pharmacophore model and its relationship with JAK family selectivity and adverse effects

Systematic analysis of reported JAK/STAT pathway inhibitors allows the summarization of their general SAR features. JAK/STAT inhibitors typically contain five major pharmacophores: a hydrophobic head, a linker (NH, O, or CH_2_), a heterocyclic core moiety, an amide bond, and a hydrophobic tail.

1. Hydrophobic head: This is often composed of heterocyclic rings such as morpholine, piperazine, substituted benzene, pyrrolidine, tetrahydroquinoline, thiomorpholine, or thiadiazole. These groups form hydrophobic interactions and hydrogen bonds with conserved residues in the ATP-binding pocket of JAK kinases (e.g., Asp912, Lys677, Lys943, Val836), determining the baseline affinity for different JAK subtypes (JAK1, JAK2, JAK3, TYK2). For example, the piperidine ring of tofacitinib forms a specific interaction with Cys909 of JAK3, enhancing its selectivity for JAK3, whereas the pyrazolopyrimidine scaffold of baricitinib adapts to the “gatekeeper” residue Met929 of JAK2, conferring JAK1/2 selectivity.
2. Linker (NH, O, or CH_2_): This connects the hydrophobic head to the heterocyclic core, and its length and flexibility affect the overall molecular conformation. This region interacts with amino acid residues such as Pro903, Asn140, Leu932, and Met665. Shortening the linker or introducing a rigid structure (e.g., CH_2_) can reduce conformational flexibility, thereby improving selectivity for a specific JAK subtype.
3. Heterocyclic core moiety: This is the key part for inhibitor binding to the kinase hinge region. Common cores include pyrimidine, pyridine, pyrazole, pyrrole, indole, triazole, pyridazine, azepine, pyrazine, and imidazole. These heterocycles form hydrogen bonds with hinge-region residues such as Leu905, Tyr904, Ala853, Val863, and Ser704. Substitution patterns on the core (e.g., fluorine, methyl) can significantly alter electron distribution and steric hindrance, thereby modulating inhibitor potency and subtype selectivity. For instance, deucravacitinib adopts a unique pseudokinase (JH2) allosteric binding mode that does not directly compete for the ATP site, resulting in very high TYK2 selectivity and theoretically reducing the peripheral immunosuppressive and anemic adverse effects associated with broad-spectrum JAK inhibition.
4. Amide bond: This connects the heterocyclic core to the hydrophobic tail. It generally helps maintain a linear molecular conformation, and its carbonyl and NH groups can form hydrogen bonds with surrounding amino acids, stabilizing the inhibitor-kinase binding mode.
5. Hydrophobic tail: This typically contains piperazine, cyclobutene, cyclopropane, morpholine, substituted benzene, or hydrocarbon chains. Substituents on the hydrocarbon chain, such as methyl, trifluoromethyl, acetyl, cyano, or amino groups, engage in hydrophobic packing or polar interactions with residues like Asp976, Ser936, and Val863, directly affecting metabolic stability and plasma protein binding.

Currently, researchers are developing heterocyclic scaffolds, including pyrimidine, pyrazole, pyridine, imidazole, indole, and sulfonamide derivatives. Specifically, compound 161 (pyrimidine core) showed high enzymatic inhibitory activity against JAK2 (IC_50_ = 0.5 μM). Compound 230 exhibited excellent inhibitory activity against JAK3 (IC_50_ = 0.4 nM), outperforming the reference molecules ibrutinib and tofacitinib. The cyano-substituted pyrimidine derivatives compound 190 and compound 191 showed IC_50_ values of 22.86 nM and 20.66 nM against JAK, comparable to tofacitinib (IC_50_ = 20.10 nM). Against JAK2, compounds 155, 178b, 205, and 252 were identified as the most potent candidates, with IC_50_ values of 2.01 nM, 3.6 nM, 1.8 nM, and 3 nM, respectively. Against JAK3, compounds 170, 178b, 220, and 225 gave IC_50_ values of 2.0 nM, 3.8 nM, 1.38 nM, and 1.7 nM, respectively. Against JAK1, compounds 178a, 200a, 200b, and 274 showed IC_50_ values of 0.3 nM, 33.4 nM, 19.9 nM, and 6 nM, respectively. These data demonstrate that fine tuning of the heterocyclic core and side-chain substituents can achieve marked selectivity differences among JAK subtypes.

Furthermore, the Val617Phe (V617F) mutation in the JH2 domain of JAK2 is the most common acquired mutation in myeloproliferative neoplasms. This finding suggests that selective inhibitors targeting mutant JAK2 may have dual value in patients with both tumors and NDDs. Although such mutations are not common in classical NDDs, this example highlights the strategy of designing highly selective inhibitors based on specific pathological mutations. In the future, systematic optimization of the five pharmacophores described above should lead to high-selectivity inhibition of JAK subtypes, which is a prerequisite for reducing adverse effects such as infections, anemia, and neoplasms during long-term therapy.

#### 5.1.2 Challenges in drug repurposing and the BBB

The BBB strictly regulates the passage of substances between the blood and brain tissue (Montagne et al., 2017); the tightly junctional endothelial cells of the cerebral microvasculature, the basement membrane, and the astrocyte processes collectively form its structural barrier. In terms of substance selectivity, this barrier permits only small, lipophilic molecules or certain substances transported via active transport mechanisms to pass through, while most polar molecules and exogenous drugs are blocked from entering (Alavijeh et al., 2005). Furthermore, active efflux transporters are widely present at the BBB; these can pump compounds that have entered the brain parenchyma back into the bloodstream, thereby reducing their exposure and residence time in the brain and consequently limiting the manifestation of their pharmacological effects.

To investigate the potential off-target effects of the JAK inhibitor baricitinib in AD, Faquetti et al. employed a multidisciplinary integrated strategy and found that baricitinib inhibits two kinases associated with AD pathology: CK2-α2 (Kd = 5.8 μM) and *MAP3K12* (Kd = 5.8 μM) (Faquetti et al., 2024). Excessive activation of the former is associated with abnormal tau phosphorylation and Aβ generation in AD (Greenwood et al., 1994; Masliah et al., 1992), while the latter, as a key regulator of neurodegeneration in the JNK pathway, may selectively mediate neuronal degeneration and apoptosis when inhibited (Clewell et al., 1999; Patel et al., 2015). Although baricitinib was previously mentioned as having multiple neuroprotective mechanisms, its inhibitory effects at the molecular level and the BBB’s low permeability to the drug may make it difficult for actual intracerebral drug concentrations to produce effective pharmacological effects. This reflects the limited potential for successful drug repurposing in AD (Faquetti et al., 2024); therefore, whether the selective permeability of the BBB can be reasonably addressed is a key issue in NDDs drug development (Alavijeh et al., 2005; Ball et al., 2013). Furthermore, for neurotherapeutic drugs to exert their pharmacological effects, they must not only cross the BBB but also remain in the brain for a sufficient duration. However, brain transporters (such as P-glycoprotein) can clear drugs from the CNS, which also reduces drug bioavailability to some extent (Gil-Martins et al., 2020; Chai et al., 2022). In response, increasing plasma drug concentrations can allow some drugs to achieve a certain level of brain exposure; however, this often exceeds the doses predicted by *in vitro* affinity studies, thereby increasing the risk of systemic toxicity. Early epidemiological studies found that individuals taking high-dose aspirin had a lower incidence of AD and improved cognitive maintenance (Nilsson et al., 2003). However, a recent controlled study showed an increased incidence of bleeding in patients taking aspirin (Jaturapatporn et al., 2012). Consequently, drugs intended for peripheral conditions—such as the JAK inhibitor baricitinib—often exhibit compromised efficacy when applied to CNS diseases like AD due to their low BBB permeability and significant efflux effects.

In a recent groundbreaking, mechanism-driven, prospective early-phase clinical trial (NCT05189106), investigators administered the JAK inhibitor baricitinib to participants using a dose-escalation regimen to evaluate the therapeutic potential of this neuroinflammation-targeting drug in NDDs. Key objectives of the study included determining whether baricitinib could achieve therapeutic concentrations in cerebrospinal fluid (CSF) and reduce levels of inflammatory biomarkers in the CSF of individuals with AD or those at high risk. If positive results are obtained, subsequent studies will focus on establishing the optimal dose and treatment duration, exploring the use of blood biomarkers as an alternative to CSF testing, and validating its efficacy in slowing the progression of AD through Phase III clinical trials, as well as expanding its application to other brain diseases characterized by neuroinflammation.

From a broader perspective, the distribution of current clinical trials targeting the JAK/STAT pathway in NDDs exhibits significant disparities. In the field of MS, drugs such as natalizumab have accumulated the most extensive clinical trial data, spanning multiple phases from Phase 2 to Phase 4 as well as numerous observational studies. These studies primarily focus on outcome measures such as annualized relapse rate, changes in MRI lesions, drug safety, and patient preference. However, the number of interventional trials currently recruiting patients in this field is relatively limited; most studies have been completed or are active but no longer recruiting. In the AD field, several Phase 3 trials, including TTP488, have been terminated. This was not due to safety concerns, but rather because the 5 mg dose failed to demonstrate sufficient efficacy. Although the Phase 1/2 basket trial of baricitinib in AD and ALS (NCT05189106) has been completed, its primary outcomes focused on cerebrospinal fluid drug concentrations and inflammatory biomarkers, constituting exploratory studies on safety and pharmacokinetics; larger-scale efficacy validation studies are still needed. In the field of ALS, the early Phase 1 trial of tofacitinib (NCT06689982) has not yet begun patient recruitment, and the overall research remains in its initial stages. The current status described above indicates that clinical validation of the JAK/STAT pathway in NDDs is still in an early or uneven stage of development, and the barrier posed by the BBB to central nervous system drug delivery is one of the key bottlenecks limiting further clinical translation (Table 4).

**Table 4:** Clinical trials of JAK/STAT-targeting agents in neurodegenerative diseases.

Type of disease	Clinical trial number	Drugs	Research stage	Primary outcome measures	State
AD	NCT05189106	Baricitinib	Phase 1/2	CSF drug concentrations, inflammatory biomarkers.	Completed
	NCT02080364	TTP488	Phase 3	Changes in AD cognition and clinical dementia rating scores compared to baseline.	Terminated
	NCT02916056	TTP488	Phase 3	Number of patients who experienced at least one adverse event.	Terminated
	NCT03980730	TTP488	Phase 3	Change from baseline on the AD Assessment of Cognitive Subscale (ADAS-cog14).	Terminated
	NCT04529876	Tofacitinib	Observational	Onset of dementia and Alzheimer’s disease.	Completed
	NCT04691505	Hydroxychloroquine/Methotrexate	Observational	Onset of dementia and Alzheimer’s disease.	Completed
MS	NCT05418010	Natalizumab	Phase 2	Change in the mean magnetization transfer ratio of FLAIR-high-signal lesions at 12 weeks compared to baseline.	Recruiting
	NCT04115488	Natalizumab	Phase 3	Total number of new active lesions within 24 weeks.	Completed
	NCT03689972	Natalizumab	Phase 3	At the end of the crossover period, the proportion of participants who preferred subcutaneous administration of natalizumab.	Completed
	NCT03135249	Natalizumab	Phase 4	Annualized recurrence rate since discontinuation of natalizumab treatment.	Completed
	NCT03516526	Natalizumab	Phase 4	Gadolinium-enhancing T1 lesions on brain MRI.	Completed
	NCT04178005	Natalizumab	Phase 4	Absolute and percentage changes in T cells, B cells, DC subsets, and NfL levels in the blood.	Active, not recruiting
	NCT03535298	Natalizumab	Phase 4	Brain volume loss from baseline to month 36.	Active, not recruiting
	NCT04777539	Natalizumab	Observational	Number of Grade 2 serious adverse events and selected adverse events.	Completed
	NCT05925049	Natalizumab	Observational	The proportion of participants who began receiving natalizumab and developed antibodies.	Active, not recruiting
	NCT03399981	Natalizumab	Observational	Number of participants diagnosed with progressive multifocal leukoencephalopathy.	Completed
	NCT05627271	Natalizumab	Observational	Number of participants with key symptoms associated with the fading effect.	Completed
	NCT02965170	Natalizumab	Observational	Serum natalizumab concentration.	Completed
	NCT03193866	Natalizumab	Observational	Patients with a Modified Disability Status Scale score <2.5 and an EDSS ≥2.5 at baseline were diagnosed with disease progression.	Completed
	NCT05236777	Natalizumab	Observational	Number of participants with progressive multifocal leukoencephalopathy who experienced serious adverse events while taking natalizumab.	Completed
	NCT05304520	Natalizumab	Observational	Number of participants at Month 6, based on their preferred natalizumab administration route.	Completed
	NCT04580381	Natalizumab	Observational	Annual recurrence rate among patients.	Completed
	NCT05209815	Natalizumab	Observational	The proportion of patients who experienced serious adverse events during pregnancy.	Completed
ALS	NCT05189106	Baricitinib	Phase 1/2	CSF drug concentrations, inflammatory biomarkers.	Completed
	NCT06689982	Tofacitinib	Early Phase 1	Changes in ALSFRS-R scores among ALS patients.	Not yet recruiting

Given the limitations imposed by the BBB on the utilization of neurotherapeutic drugs, future research must focus on developing new strategies to overcome this barrier. These may include circumventing efflux transporters, utilizing endogenous transport mechanisms, or constructing novel drug delivery systems to enhance intracerebral bioavailability. JAK inhibitors that achieve high selectivity, good BBB penetration, and suitable PK properties through the SAR optimization strategies described above hold promise for achieving true disease modification in NDDs models, rather than merely providing short-term symptomatic relief.

### 5.2 JAK/STAT inhibitors in NDDs: challenges in clinical translation

Although extensive preclinical studies have demonstrated the efficacy of JAK/STAT inhibitors in models of neuroinflammation, their limitations become apparent when these data are re-evaluated against the rigorous standards of clinical translation.

Take the study of baricitinib in an ovariectomy/D-galactose-induced AD rat model as an example. Although the study confirmed its beneficial effects on neuroinflammation, A*β* plaque accumulation, and neuronal death, the dosing regimen was prophylactic or early intervention, failing to simulate the extensive pathological damage that may already be present at the time of patient presentation (Hindam et al., 2024). Similarly, tofacitinib was also administered acutely in an LPS-induced AD model (Yang et al., 2021), with endpoints limited to short-term improvements in spatial memory and reduced levels of pyroptosis, failing to assess the true impact of long-term treatment on disease progression. Furthermore, in an *α*-synuclein-induced PD model, although AZD1480 demonstrated multiple effects in inhibiting microglial activation and T-cell infiltration (Qin et al., 2016), the timing of intervention was similarly concentrated in the acute inflammatory phase, and there is a lack of validation regarding the persistence of efficacy during the chronic course of the disease.

A common feature of the aforementioned cases is that interventions are largely limited to prophylactic dosing or short-term regimens, which falls far short of the clinical reality that patients with NDDs require long-term treatment and already have irreversible pathological damage. At the same time, the design of most current drugs does not fully account for the CNS penetration, and there is a lack of key data such as intracerebral drug concentrations, long-term follow-up, and comparisons of treatment effects. Furthermore, existing studies have not explored potential issues such as drug resistance, STAT feedback upregulation, or diminishing efficacy under long-term use. This makes it difficult to determine whether these inhibitors can truly achieve “disease modification”—that is, delaying or reversing the progression of neurodegenerative lesions—or whether they merely provide temporary symptom relief. The former is the core objective that requires primary focus in the treatment of NDDs.

Regarding the safety concerns associated with long-term use of JAK inhibitors, long-term safety data for tofacitinib and baricitinib in peripheral immune disorders such as RA provide important warnings. Long-term follow-up results indicate that infections are the most common adverse events associated with long-term use of these two drugs; other serious adverse events include pneumonia, herpes zoster, malignancies, and gastrointestinal perforation, among others (Cohen et al., 2020; Taylor et al., 2022). However, current preclinical research in the field of NDDs pays little attention to these safety concerns. The vast majority of studies report only short-term efficacy metrics and have not systematically evaluated the risks of immunosuppression, opportunistic infections, and tumorigenesis that may be induced by long-term medication use.

Furthermore, it is important to note that data from patients with RA have clearly demonstrated that advanced age is a significant risk factor for infections. Since patients with NDDs (such as AD and PD) are predominantly elderly, their baseline immune function naturally declines with age. Combined with the common practice of polypharmacy, this further amplifies the risk of adverse reactions associated with JAK inhibitors. Therefore, when prescribing JAK inhibitors to elderly patients with NDDs, the potential long-term adverse risks must be weighed equally against the therapeutic benefits, and clinical translation should not be pursued based solely on short-term preclinical studies.

### 5.3 From neuroinflammation, neuroprotection to neuroregeneration: new therapeutic directions for the JAK/STAT pathway

As discussed earlier, the activation of microglia and astrocytes has a dual effect: under classical activation pathways (such as the IFN-*γ*/LPS–NF-κB/STAT1 signaling axis), both cell types can be induced into a reactive state, releasing pro-inflammatory factors such as IL-1β, TNF-α, and IL-6, thereby driving neuroinflammation and neurodegeneration; however, during normal brain development, they participate in establishing proper synaptic connections and maintaining normal brain function by clearing damaged cells and dysfunctional synapses (Kettenmann et al., 2013; Clarke and Barres, 2013).

Current treatments for neuroinflammation primarily focus on globally suppressing the inflammatory response; however, this approach may also weaken the body’s inherent protective mechanisms. In the field of NDDs treatment, the key to transitioning from “neuroinflammation” to “neuroprotection” and ultimately to “neurogenesis and functional recovery” lies in the effective regulation of phenotypic switching in microglia and astrocytes. Studies have shown that IL-4, IL-10, IL-13, and TGF-β can promote the transition of microglia and astrocytes from a pro-inflammatory phenotype to a neuroprotective phenotype by activating STAT3 and STAT6, thereby facilitating the release of neurotrophic factors and tissue repair (Liddelow and Barres, 2017; Tang and Le, 2016; Kwon and Koh, 2020), while also reducing the levels of pro-inflammatory factors such as IL-6, TNF-α, and NO (Kwon and Koh, 2020; Zhao et al., 2006; Park et al., 2005). Therefore, future developments should focus on drugs that precisely regulate the functional states of microglia and astrocytes, which will hold significant therapeutic value.

In recent years, a large body of research has shown that targeting specific molecules in the JAK/STAT signaling pathway can precisely regulate downstream inflammation-related events. While the development of JAK inhibitors has reached a relatively mature stage, strategies targeting STAT are showing a trend toward diversification and precision. Taking STAT3 as an example, it serves as a central node in neuroimmune regulation, playing a crucial role in NDDs mediated by reactive astrocytes, while also directly participating in the regulation of oligodendrocyte precursor cell maturation and myelin regeneration (Bao M. Y. et al., 2024).

In addition, the combination of well-established JAK inhibitors with neurotrophic factors is expected to simultaneously suppress neuroinflammation and synergistically promote the structural and functional repair of neural circuits (Gao et al., 2025; Bella et al., 2006; Khodir et al., 2024). To this end, it is imperative to address the issue of drug delivery efficiency within the brain to achieve maximum therapeutic efficacy and minimize side effects with combination therapy. Through interdisciplinary innovation, advancing JAK/STAT-targeted therapy from neuroprotection toward neuroregeneration will open new pathways for improving the clinical prognosis of NDDs.

## 6 Conclusion

The JAK/STAT signaling pathway plays a pivotal and integrative role in the pathological progression of NDDs. It directly exacerbates neuroinflammation and neuronal damage by driving BBB dysfunction, inducing reactive activation of microglia and astrocytes, and promoting the release of pro-inflammatory factors; it also contributes to abnormalities in brain energy metabolism and demyelination in MS, thereby closely linking immune dysregulation, protein homeostasis imbalance, and neurodegeneration. JAK inhibitors, represented by baricitinib, tofacitinib, and ruxolitinib, have demonstrated clear anti-inflammatory and neuroprotective effects in preclinical models; however, their clinical application is limited by bottlenecks such as low BBB permeability, the risk of systemic infection, and a lack of cell-type selectivity, making it difficult to achieve a transition from central anti-inflammation to functional restoration. The most promising future approach should shift from global inflammation suppression to the precise reprogramming of glial cell phenotypes, particularly by targeting core transcription factors such as STAT3. This involves developing highly selective small-molecule inhibitors or nucleic acid therapeutics to drive the transition of microglia and astrocytes from pro-inflammatory states to neuroprotective phenotypes. Concurrently, the combination of JAK/STAT inhibitors with neurotrophic factors or cell therapies is expected to synergistically promote the maturation of oligodendrocyte precursor cells, myelin regeneration, and neural circuit repair. Overcoming the limitations of intracerebral drug delivery efficiency and establishing reliable biomarkers of therapeutic efficacy will be key to advancing targeted therapies along this pathway from neuroprotection toward neuroregeneration and improving the clinical prognosis of NDDs.
